# The College Election

**Published:** 1920-05-22

**Authors:** 


### THE COLLEGE ELECTION.

The nursing profession in England, if the truth be told, is in its early youth. And, as S. Paul advised his friend, I would say, "let no man despise thy youth." Youth is the one time of life when romance excites the imagination, and when all things seem possible, when little things appear great, and great things small. At school boys learn their lessons, but it takes experience to teach them wisdom.

"Knowledge comes, but wisdom lingers." Hence the necessity for those who have lived longest and best to guide the nurse along the path that leads upward. In the world's transition, now in progress, there are great possibilities for the nurse. But there are also pitfalls and dangers. The world is confronted with extremely complex problems, and of these the nurse will have her share. For example, the forty-eight hours' week question is in the foreground, and the most diverse views are held concerning it, so far as its application to nurses is concerned. Some hold that it is out of the question, and those who favour it realise that it is beset with difficulty. Leaders of one school advocate the *fortiter in re* attitude, whilst those of another adopt the policy of the ostrich, and simply ignore its existence. Then there is the great question of nurses as the proper and legitimate administrators of Health reconstruction. At present, although there are according to the Regulations privileges for nurses to enter the Ministry of Health service, yet in actual fact they are debarred, and outsiders are quietly entering into possession. Greater than either of these, from the public standpoint, is the problem of the voluntary hospital, whose very existence is in danger. The pay and conditions of service of the nurse of the future will be a potent factor in its solution. If she is not permitted, like the rest of the world, to progress, she may bow her head. But the recruitment of high-class probationers will automatically stop.

I mention these things in order to show that the leaders of the profession will require all their wisdom and ability to bring about reconstruction on lines which will uplift the nurse, and at the same time enable her to hold the high position which she already occupies in the public esteem.

It is natural that one should look at so difficult a juncture for light and leading to the College of Nursing. Numerically and otherwise it is the largest Nurses' Union in the kingdom. It was established to solve war and post-war nursing problems, but, so far as I am aware, the nursing world, including College of Nursing members, knows very little as to the views held by the Executive Council, collectively and individually, concerning such vital matters as I have referred to. Equally little is known as to their activities in endeavouring to assimilate present conditions with those on which we are entering. And now we are having the annual College Election. Twelve new members are to be appointed, and a day or two after this issue of The Hospital is in circulation the voting papers will be returned.

I confess that the method of conducting this Election disappoints me. In one respect it is above reproach. In another it is singularly lacking. The elector is presented with sixty-six names, and is asked to select not more than twelve, and to signify her choice by a mark. Some of these names are familiar; some are famous. But a very large number of them are strange. And of the best known who can guess what is their attitude where nurses' interests are in question? Probably the best-known name of the whole sixty-six is that of Lord Knutsford. Speaking for myself, if I were asked to set out what policy the Chairman of the "London" would probably advocate with regard to any of the items which I have instanced, I should be utterly unable to do so. And if this applies to so distinguished a nominee, what of the lesser lights? In participating in the Election the nurse is virtually asked to take part in a lottery. She may draw a prize, or perchance a blank, or, mayhap, an enemy. Who knows?

If the nurse is to emerge, as she may, with the benefit and betterment which are her due, from the present condition of revolutionary change in the world's social and economic conditions, her leaders will find it necessary to face her problems in a business-like manner, and with concrete proposals. I had hoped that nominees from the College Executive would have anticipated this, and issued something in the nature of manifesto, so that electors would have some guide to help them in recording their votes. It is the lack of such that embarrasses and mystifies the busy hard-working nurse, and that likewise gives opportunities to other prophets to arise, such as the trade unionists, who persuade her that via their doors is the way of salvation.

